# Health inequalities in post-COVID-19 outcomes among adults aged 50+ in Europe: has COVID-19 exposed divide between postcommunist countries and Western Europe?

**DOI:** 10.1136/jech-2023-220771

**Published:** 2023-07-09

**Authors:** Maika Ohno, Dagmar Dzurova, Petr Smejkal

**Affiliations:** 1 Research Centre on Health, Quality of Life and Lifestyle in a Geodemographic and Socioeconomic Context (GeoQol), Department of Social Geography and Regional Development, Faculty of Science, Charles University, Prague, Czech Republic; 2 Infectious Disease and Infection Control, IKEM Hospital, Prague, Czech Republic; 3 Hospitalist and Infection Control, Mount Desert Island Hospital, Bar Harbor, Maine, USA; 4 1st Medical Faculty, Institute of Hygiene and Epidemiology, Charles University, Prague, Czech Republic

**Keywords:** COVID-19, DEMOGRAPHY, Health inequalities, OBESITY, GERONTOLOGY

## Abstract

**Background:**

COVID-19 affected people and countries disproportionately and continues to impact the health of people. The aim is to investigate protective health and socio-geographical factors for post-COVID-19 conditions in adults aged 50 years and older in Europe.

**Methods:**

Using longitudinal data from the Survey of Health, Ageing and Retirement in Europe, collected from June to August 2021, protective factors against post-COVID-19 condition among 1909 respondents who self-reported a positive COVID-19 test result were investigated using multiple logistic regression models.

**Results:**

Male adults living outside of Czechia, Poland, Hungary and Slovakia (Visegrad group, V4), who received the COVID-19 vaccination, tertiary or higher education, had a healthy weight (body mass index, BMI 18.5–24.9 kg/m^2^) and no underlying health condition/s, showed protective effects against post-COVID-19 condition. Health inequalities associated with BMI were observed in education attainment and comorbidities, with higher BMI having lower education attainment and higher comorbidities. Health inequality was particularly evident in individuals in V4 with higher obesity prevalence and lower attainment of higher education than those living in other regions in the study.

**Conclusion:**

Our study suggests that healthy weight and higher education attainment are predictors associated with a lower incidence of post-COVID-19 condition. Health inequality associated with education attainment was particularly relevant in V4. Our results highlight health inequality in which BMI was associated with comorbidities and educational attainment. To reduce obesity prevalence among older people with lower education, raising awareness about the risks of obesity and providing assistance in maintaining a healthy weight are needed.

**WHAT IS ALREADY KNOWN ON THIS TOPIC**

- It has been reported that people suffer from lingering symptoms after recovering from an initial infection of COVID-19 regardless of the severity of the initial infection. Characteristics of individuals with post-COVID-19 condition in older people in Europe are still not well established.

**WHAT THIS STUDY ADDS**

- This study highlights the relationship between post-COVID-19 condition and health inequality associated with obesity and education attainment in older adults.
- Healthy weight, COVID-19 vaccination and high education attainment showed protective effects against post-COVID-19 condition in people aged 50 and above.
- Health inequality was particularly evident in individuals in the Visegrad group (Czechia, Poland, Hungary and Slovakia) with higher obesity prevalence and a smaller proportion of high education attainment.

**HOW THIS STUDY MIGHT AFFECT RESEARCH, PRACTICE OR POLICY**

- This study underscores health inequality stemming from education attained at earlier life and hence the importance of reducing educational inequalities.
- Raising awareness about the risks of obesity and providing assistance in maintaining a healthy weight in older people are needed.

## Introduction

The WHO estimated that in 2020 and 2021 Europe alone had 17 million people suffered from post-COVID-19 condition, also commonly known as ‘Long-COVID’, with symptom(s) lasting at least 3 months.^1^ It has been reported that people suffer from lingering symptoms after recovering from an initial infection of COVID-19 regardless of the severity of the initial infection.^2 3^ The high-risk factors for developing severe COVID-19 symptoms are well established, which include sex, age, body mass index (BMI) (specifically male adults aged 65 and above, with a BMI above 30 kg/m^2^), and individuals with comorbidities and who are not vaccinated.^4 5^ However, evidence on subsequent risks of developing post-COVID-19 condition is still limited. Studies that investigated risk factors of developing post-COVID-19 condition showed elusive results. Higher prevalence of post-COVID-19 condition has been reported among older people, female sex and people with comorbidities,^6 7^ while a large UK population study reported that the risk of developing post-COVID-19 decreased with age as compared with younger age 18–30 years.^2^ COVID-19 vaccination has been proven to decrease risks for developing severe illnesses,^8^ however, protective effects of COVID-19 vaccination on the development of post-COVID-19 condition are equivocal. Vaccinated people who had breakthrough infections with COVID-19 had lower risk of developing pos-COVID-19 condition.^9^ Another study showed, however, no significant protective effects against post-COVID-19 condition on people with single vaccination.^10^

COVID-19 infection rates and the number of deaths varied greatly across European countries. The excess mortality rates in the Eastern European regions were among the worst as compared with other EU member states. Between November and December 2020 when the EU faced the second wave of excess mortality, the Visegrad group (V4) consisting of Poland (PL), Czechia (CZ), Slovakia (SK) and Hungary (HU) had the highest excess mortality rates, 97%, 76%, 58% and 59%, respectively.^11^ One year later, EU member states had between none to 20% excess mortality between October and November 2021, in comparison to 42%–73% among the V4 group.^11^ To our knowledge, little is known about prevalence of post-COVID-19 condition in the V4 group.

Our previous study investigating severity of COVID-19 outcomes in older subjects aged 50 and above across European countries showed that the likelihood of hospitalisation increased with age and BMI, and among unvaccinated people.^12^ However, protective factors for post-COVID-19 condition in the same population and geographical disparities in post-COVID-19 condition are little known. Therefore, the aims of our study are, first to assess prevalence of post-COVID-19 condition among people aged 50 and above who had a positive COVID-19 test; second, to assess protective factors against post-COVID-19 condition; and lastly, to assess whether protective factors differ between V4 and other countries.

## Methods

### Study design and participants

The Survey of Health, Ageing and Retirement in Europe (SHARE), established in 2004, aims to study the life of people aged 50 and older across 28 European countries and Israel. Longitudinal data are collected every 2 years, and the methodology of SHARE has been described previously.^13 14^ Datasets used in our study were from wave 8 and wave 9 (COVID-19 survey 2) obtained from an online data catalogue of the SHARE Research Data Centre.^15 16^ Before the outbreak of COVID-19, the survey was conducted via face-to-face interviews, however, the face-to-face interviews were suspended during wave 8 in March 2020 due to the outbreak of COVID-19. Subsequently, regular wave 8 transitioned to Computer-Assisted Telephone Interview (CATI), the SHARE Corona survey 1 from June to August 2020, followed by wave 9 SHARE Corona survey 2 from June to August 2021, also conducted by CATI. Questions in both SHARE Corona surveys 1 and 2 consisted of COVID-19-related questions and changes in life during the COVID-19 pandemic.

**Figure 1:**
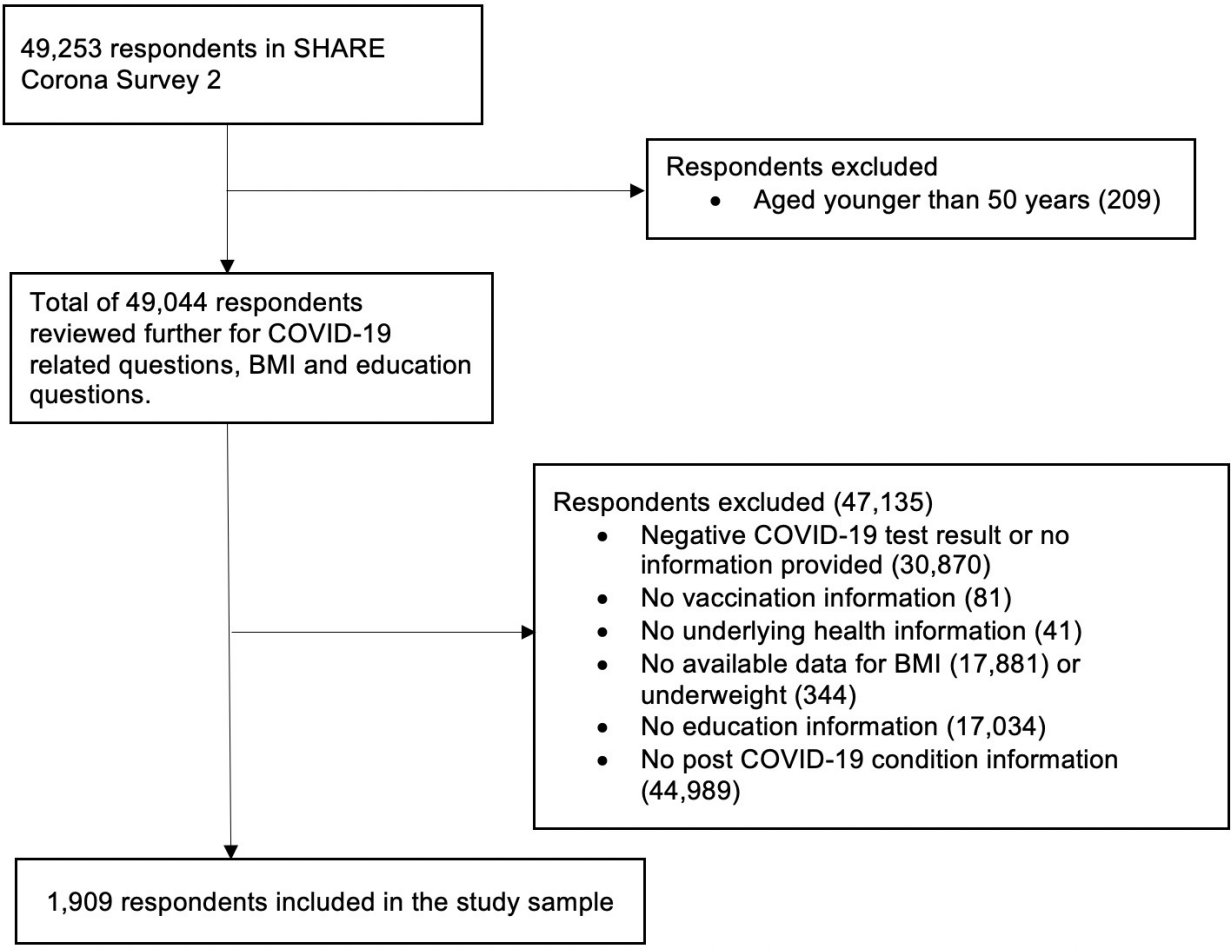
Flow chart showing selection of study sample. BMI, body mass index; SHARE, Survey of Health, Ageing and Retirement in Europe.

For our study, respondents aged 50 years and older who participated in wave 8 and wave 9 SHARE Corona survey 2, with no missing values for the selected variables were included in our analyses (figure 1). Wave 8 was used for information on education and BMI, which were not included in wave 9 SHARE Corona survey 2. Since the aim of our study was to investigate protective factors against post-COVID-19 condition, respondents who did not have COVID-19 were excluded. Of the total 49 253 respondents, 47 344 were excluded for at least one of the following reasons: (1) negative COVID-19 test result or no test information; (2) no vaccination information; (3) no information on underlying health condition; (4) no information on BMI or being underweight (BMI≤18.5 kg/m^2^); (5) no education information and (6) no valid information on post-COVID-19 condition. This resulted in the study sample of 1909 respondents from 27 European countries (Portugal was excluded due to missing BMI information in wave 8) plus Israel. Respondents with underweight (N=17) which accounted for only 0.9% of the study sample were excluded in the analysis.

### Measures

The primary outcome in our study was to investigate protective factors against post-COVID-19 condition among those who tested positive for COVID-19. COVID-19 infection cases were defined as answering ‘yes’ to the question ‘*Have you or anyone close to you been tested for the Corona virus and the result was positive, meaning that the person had the Covid disease?’*, and choosing the answer *‘1. Respondent’* for the subsequent *question ‘Who was tested positive? Please tell me their relationship to you’.* The prevalence of post-COVID-19 condition was measured using the following question in the survey: ‘*Have you experienced any long-term or lingering effects that you attribute to your Covid illness?’*. Respondents were asked to select or not select the following condition(s): (1) fatigue, (2) cough, congestion, shortness of breath, (3) loss of taste or smell, (4) headache, (5) body aches, joint pain, (6) chest or abdominal pain, (7) diarrhoea, nausea, (8) confusion and (9) other. Respondents were dichotomised into the following groups: (A) respondents who did not select any of the conditions (no condition) and (B) respondents who selected at least one of the conditions (at least one condition).

### Exposures

A total of seven explanatory variables, four sociodemographic variables (age, sex, education and region) and three health-related variables (BMI, underlying health conditions and COVID-19 vaccination uptake) were included in our binary logistic regression. Classification of education, BMI categories, underlying health conditions, COVID-19 vaccination and regions are explained below.

### Age

Age was treated as a continuous variable.

### Education

Education was measured based on ISCED-97 (International Standard Classification of Education) codes obtained from wave 8 data. The ISCED-97 levels were aggregated into three groups used by Eurostat: low (ISCED-97 levels 0–2=below lower secondary or second stage of basic education), medium (ISCED-97 levels 3–4=upper secondary or postsecondary non-tertiary education) and high (ISCED-97 levels 5–6=first stage of tertiary education or higher)^17^

### BMI categories

BMI (kg/m^2^) was obtained from wave 8 and categorised into four standard categories defined by the WHO classification: underweight (<18.5 kg/m^2^), healthy weight (18.5–24.9 kg/m^2^), overweight (25–29.9 kg/m^2^) and obesity (≥30 kg/m^2^).

### Underlying health conditions

Comorbidity was assessed using the question ‘Do you have any of the following illnesses or health conditions? Please answer yes or no to each.

Category: (1) Hip fracture? (2) Diabetes or high blood sugar? (3) High blood pressure or hypertension? (4) A heart attack including myocardial infarction or coronary thrombosis or any other heart problem including congestive heart failure? (5) Chronic lung disease such as chronic bronchitis or emphysema? (6) Cancer or malignant tumour, including leukaemia or lymphoma, but excluding minor skin cancers? (7) Any other illness or health condition?’. For binary logistic regression, respondents were dichotomised into the following groups: (A) respondents who did not select any of the conditions and (B) respondents who selected at least one of the conditions.

### COVID-19 vaccination

Respondents’ COVID-19 vaccination status was determined using the question ‘Have you been vaccinated against COVID-19?’. Respondents were defined as having been vaccinated when they answered ‘yes’ to the question and dichotomised into a vaccinated group and a not-vaccinated group.

### Region

European countries and Israel from the SHARE study were dichotomised into V4 and non-Visegrad group (non-V4). Countries included in V4 were CZ, HU, PL and SK. V4 was formed in 1991 to cooperate to overcome shared struggles after the collapse of communism in the Central Europe. During the survey period, the countries in V4 had much higher excess mortality rates due to COVID-19 than other European countries.^11^ Non-V4 included: Austria, Belgium, Bulgaria, Croatia, Cyprus, Denmark, Estonia, Finland, France, Germany, Greece, Italy, Latvia, Lithuania, Luxembourg, Malta, The Netherlands, Romania, Slovenia, Spain, Sweden, Switzerland and Israel.

### Statistical analysis

Statistical analyses were performed using IBM SPSS V.28. Descriptive statistics summarise sociodemographic and health-related characteristics of the study sample according to post-COVID-19 condition. Categorical variables were expressed in percentages. Mean and SD were used for continuous variables. Baseline characteristics of the study sample were compared based on post-COVID-19 condition using a χ^2^ test. Binary logistic regression was performed to assess whether having no post-COVID-19 condition was associated with sociodemographic factors (sex, age, education and region) and health-related factors (BMI, underlying health and COVID-19 vaccine uptake). Our binary logistic regression models included four models with a binary outcome variable being no post-COVID-19 condition among those who were tested COVID-19 positive. Respondents who reported at least one post-COVID-19 symptom were coded as 0 while respondents who reported no post-COVID-19 symptom were coded as 1. ORs with 95% CIs were estimated between individuals without post-COVID-19 condition and those with post-COVID-19 condition.

#### Model 0 (unadjusted model, crude)

Each exposure variable was entered separately to explore unadjusted association between no post-COVID-19 condition and each exposure.

#### Model 1

ORs were adjusted for sex (reference=Female), age (continuous) and education levels (reference=low).

#### Model 2

BMI (reference=obesity), comorbidity (reference=with at least one underlying health condition) and COVID-19 vaccination status (reference=not vaccinated) were added to model 1.

#### Model 3

Regions V4 (reference) and non-V4 were added to Model 2.

Differences in BMI were examined across education levels (low, medium and high) and the number of underlying health conditions (none, one condition, two conditions and three or more conditions) using one-way analysis of variance (ANOVA) followed by Tukey’s post hoc test. Lastly, in order to investigate potential geographical differences that may have contributed to post-COVID-19 condition, the sociodemographic and health-related variables between V4 and non-V4 were compared using Pearson’s χ^2^ tests, and the strength of association was assessed using Cramer’s V. Significance at p<0.05 was assumed. The datasets used for the analyses are available at http://www.share-project.org/special-data-sets/share-corona-survey-2.html.

## Results

### Prevalence of post-COVID-19 condition

Descriptive characteristics of the study sample according to post-COVID-19 condition are summarised in table 1. Among the study samples who self-reported a positive COVID-19 test, 73% (N=1399) experienced at least one post-COVID-19 condition. No statistically significant age difference was observed between the groups. A higher proportion of females (77%) reported post-COVID-19 condition than males (69%). A higher proportion of the study sample without post-COVID-19 condition completed high education (27%) as compared with those with post-COVID-19 condition (18%). Higher prevalence of overweight or obesity (75%) was observed in respondents with post-COVID-19 condition than those without post-COVID-19 condition (64%). Most respondents (80%) who experienced post-COVID-19 condition had at least one underlying health condition. A higher vaccination rate (70%) was observed in respondents without post-COVID-19 condition than those with post-COVID-19 condition (63%). Most respondents in V4 (84%) experienced post-COVID-19 condition as compared with 70% in non-V4.

**Table 1:** Characteristics of study sample by post-COVID-19 condition

	No condition		At least one condition		
	(N=510)		(N=1399)		
	N	(%)	N	(%)	P values
**Sex**					
Male	246	48.2	536	38.3	<0.001
Female	264	51.8	863	61.7	
**Age group (years)**					
50–59	82	16.1	206	14.7	0.53
60–69	215	42.2	615	44	
70–79	160	31.4	409	29.2	
80+	53	10.4	169	12.1	
**Age (years), Continuous variable**	Mean	±SD	Mean	±SD	
	68.7	8.4	68.9	8.8	
**Education**					
Low (0–2)	135	26.5	426	30.5	<0.001
Medium (3-4)	239	46.9	722	51.6	
High (5-6)	136	26.7	251	17.9	
**BMI categories (kg/m** ^**2**^ **)**					
Healthy weight	182	35.7	353	25.2	<0.001
Overweight (25–29.9)	198	38.8	595	42.5	
Obese (30)	130	25.5	451	32.2	
**BMI Continuous variable**	Mean	±SD	Mean	±SD	
	27.4	4.6	28.4	4.9	
**Underlying health conditions**					
Yes	352	69	1123	80.3	<0.001
No	158	31	276	19.7	
**COVID-19 Vaccination**					
Yes	355	69.6	876	62.6	0.005
No	155	30.4	523	37.4	
**Region**					
Non-Visegrad group	435	85.3	1019	72.8	<0.001
Visegrad group	75	14.7	380	27.2	

p<0.05 is considered significant.

BMI, body mass index.

### Protective factors against post-COVID-19 condition

#### Sociodemographic factors

As presented in table 2, the unadjusted model (model 0) indicated that being male, having higher education, healthy weight, no underlying health condition, living in non-V4 showed higher protective effects against post-COVID-19 condition. In our subsequent model, after adjusting for sociodemographic factors in model 1, being male compared with female was a significant protective factor for post-COVID-19 condition (OR 1.52, 95% CI 1.24 to 1.87). Having high education compared with low education was a significant protective factor (OR 1.66, 95% CI 1.23 to 2.22).

**Table 2:** Adjusted ORs and 95% CIs for protective factors against post-COVID-19 condition

	Model 0 (unadjusted)		Model 1†		Model 2‡		Model 3§	
	OR	95% CI	OR	95% CI	OR	95% CI	OR	95% CI
**Sex**								
Female (ref)								
Male	1.50**	(1.22 to 1.84)	1.52**	(1.24 to 1.87)	1.57**	(1.27 to 1.94)	1.54**	(1.24 to 1.90)
**Age (continuous)**	1	(0.99 to 1.01)	1	(0.99 to 1.01)	1	(0.99 to 1.01)	1	(0.99 to 1.01)
**Education**								
Low (0–2) (ref)								
Medium (3–4)	1.05	(0.82 to 1.33)	0.98	(0.76 to 1.27)	0.97	(0.75 to 1.25)	1.04	(0.80 to 1.36)
High (5–6)	1.71**	(1.29 to 2.27)	1.66**	(1.23 to 2.22)	1.46*	(1.08 to 1.98)	1.42*	(1.05 to 1.92)
**BMI**								
Obesity (ref)								
Healthy weight	1.79**	(1.37 to 2.33)			1.53*	(1.16 to 2.02)	1.46*	(1.11 to 1.93)
Overweight	1.15	(0.90 to 1.49)			1.03	(0.79 to 1.33)	1	(0.77 to 1.30)
**Underlying health condition**								
With underlying health condition(s) (ref)								
No underlying health condition	1.83**	(1.45 to 2.30)			1.72**	(1.35 to 2.19)	1.68**	(1.31 to 2.15)
**Vaccination**								
Not vaccinated (ref)								
Vaccinated	1.37*	(1.10 to 1.70)			1.32*	(1.05 to 1.65)	1.39*	(1.11 to 1.75)
**Region**								
Visegrad group (ref)								
Non-Visegrad group	2.16**	(1.65 to 2.84)					2.03**	(1.53 to 2.69)

*p<0.05; **p<0.001.

p<0.05 is considered significant. The Visegrad group (V4) consists of CZ, HU, PL and SK.

† Model 1: Adjusted for sex, age and education.

‡ Model 2: Adjusted for sex, age, education, BMI, underlying health conditions and vaccination.

§ Model 3: Adjusted for sex, age, education, BMI, underlying health conditions, vaccination and region.

BMI, body mass index; CZ, Czechia; HU, Hungary; PL, Poland; SK, Slovakia.

#### Health-related factors

Subsequently, we examined protective effects of health-related covariates by adding variables BMI, comorbidity and COVID-19 vaccination uptake to model 1. Model 2 showed that being male (OR 1.57, 95% CI 1.27 to 1.94) and having higher education (OR 1.46, 95% CI 1.08 to 1.98) remained associated with higher protective effects against post-COVID-19 condition. As compared with obesity, the odds for healthy-weight individuals without post-COVID-19 condition were 1.5 times as large (95% CI 1.16 to 2.02). However, overweight was not a predictor for no post-COVID-19 condition. Individuals without any underlying health condition had 1.7 times greater odds for having no post-COVID-19 condition (95% CI 1.35 to 2.19) as compared with those with comorbidities. COVID-19 vaccination showed higher protective effects against post-COVID-19 condition (OR 1.32; 95% CI 1.05 to 1.65). High education attainment remained a significant predictor for having no post-COVID-19 condition.

#### Region

In our fully adjusted model (model 3), the geographical predictor variable was added to model 2 to examine the effect of the country of residence. In the fully adjusted model, non-V4, as compared with V4, was associated with higher protective effects against post-COVID-19 condition, twice as much as V4 (95% CI 1.53 to 2.69). In the fully adjusted model, the protective effects of being male, having high education, healthy weight, no underlying health condition and the COVID-19 vaccination were consistent.

#### Characteristics of BMI across education groups and the number of comorbidities: implication of health inequality

**Figure 2:**
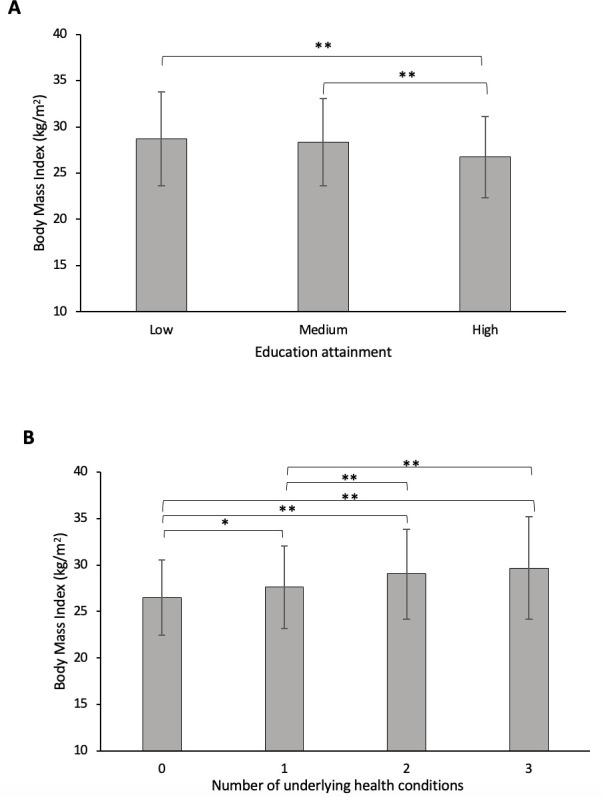
(A) Differences in BMI across different education groups (Low, Medium and High). (B) Differences in BMI by the number of underlying health conditions (0, 1, 2 and 3 or more conditions). *p<0.01, **p<0.001. p<0.05 is considered significant. Error bars represent SD. BMI, body mass index.

Figure 2 shows differences in BMI across different education groups (Low, Medium and High) and the number of underlying health conditions (0, 1, 2 and 3 or more conditions).

A one-way ANOVA revealed statistically significant differences in BMI among different education levels (*F*(21.906)=20.968, p<0.001). A Tukey’s post hoc test indicated that mean BMI was significantly lower in high education (26.7 kg/m^2^± 4.4) as compared with medium education (28.3 kg/m^2^± 4.7, p<0.001) and low education (28.7 kg/m^2^± 5.1, p<0.001). The post hoc tests also revealed significant differences in BMI associated with the number of comorbidities (*F*(3, 1905)=38.215, p<0.001). Mean BMI for the group with no underlying health condition (26.5 kg/m^2^± 4.0) was significantly lower than all the other groups (1 condition (27.6 kg/m^2^± 4.5), 2 conditions (29.0 kg/m^2^± 4.8) and 3 or more conditions (29.7 kg/m^2^± 5.5) (p<0.001). BMI increased linearly as the number of comorbidities increased. BMI for one underlying health condition was significantly lower than BMI for two conditions and three or more conditions (p<0.001). No significant difference in BMI was observed between BMI for two conditions and BMI for three or more underlying health conditions (p = 0.21).

#### Characteristics of V4 and non-V4

To assess factors that may have contributed to differences in the protective effects against post-COVID-19 condition in non-V4 as compared with V4, we measured the strength of association between V4 and non-V4 using Pearson’s χ^2^ tests and the Cramer’s V. Differences between the two groups are shown in table 3. The Pearson’s χ^2^ test with Cramer’s V revealed statically significant differences in education levels, BMI and COVID-19 vaccination between V4 and non-V4 (p<0.001). The strength of association between education and regions was moderate (Cramer’s V = 0.18). Though statistically significant, the strength of association between BMI and regions (Cramer’s V = 0.09) as well as between vaccination and regions were weak (Cramer’s V = 0.09). There was no statistically significant difference in sex across the countries (p = 0.21, Cramer’s V = 0.03).

**Table 3:** Characteristics of Visegrad and non-Visegrad countries

	Non-Visegrad		Visegrad		P value	χ2 (df)	Cramer’s V
	(N=1454)		(N=455)				
	N	(%)	N	(%)			
**Sex**							
Male	607	41.7	175	38.5	0.21	1.55(1)	0.03
Female	847	58.3	280	61.5			
**Age group (years)**							
50–59	82	16.1	206	14.7	0.08	6.63(3)	0.06
60–69	215	42.2	615	44			
70–79	160	31.4	409	29.2			
80+	53	10.4	169	12.1			
**Age, years**	Mean	±SD	Mean	±SD			
	68.7	8.4	68.9	8.8			
**Education**							
Low (0–2)	450	30.9	111	24.4	<0.001*	60.663(2)	0.18
Medium (3–4)	664	45.7	297	65.3			
High (5–6)	340	23.4	47	10.3			
**BMI categories (kg/m** ^**2**^ **)**							
Healthy weight	438	30.1	97	21.3	<0.001*	15.173(2)	0.09
Overweight (25–29.9)	597	41.1	196	43.1			
Obese (30)	419	28.8	162	35.6			
**BMI continuous variable**	Mean	±SD	Mean	±SD			
	27.4	4.6	28.4	4.9			
**Underlying health conditions**							
Yes	1108	76.2	367	80.7	0.05	3.917(1)	0.05
No	346	23.8	88	19.3			
**COVID-19 vaccination**							
Yes	904	62.2	327	71.9	<0.001*	14.222(1)	0.09
No	550	37.8	128	28.1			

*p<0.05 is considered significant.

BMI, body mass index.

## Discussion

This study examined sociodemographic and health-related characteristics of people aged 50 and above who reported a positive COVID-19 test result, but did not experience post-COVID-19 condition. In this study, respondents with healthy weight had a 1.5 times higher likelihood of having no post-COVID-19 condition as compared with respondents with obesity. Our finding supports the large cohort and longitudinal studies in which individuals with obesity had an increased risk of experiencing post-COVID-19 symptoms.^2 7 18^ We observed differences in the protective effects of healthy weight when compared with overweight and obesity. Specifically, the protective effects of healthy weight were significantly higher as compared with obesity, but not compared with overweight, which suggests a fine line between overweight and obesity. This finding is congruent with a study in which individuals with BMI ≥ 35 kg/m^2^ had higher risks of both hospital admission and more tests for medical problems by more than 25% after recovery from the infection compared with individuals with healthy weight while no significant difference was observed between overweight and healthy weight individuals.^19^ Obesity is a multifactorial disease attributed to environmental and genetic factors, leading to chronic diseases such as cardiovascular diseases, diabetes and kidney diseases.^20^ These finding conveys an important difference between overweight and obesity, and underscores the importance to maintain weight below obesity levels.

### Implications for health and social inequality

Our study indicated complex interactions between obesity, comorbidity, education attainment and post-COVID-19 condition. The COVID-19 pandemic has affected individuals across different socioeconomic groups disproportionally and exacerbated preexisting social and health inequalities.^21^ In the USA, higher mortality rates due to COVID-19 were associated with lower education and black residents.^22^ Lower educational attainment has been recognised to increase health inequality.^23^ Our study showed that COVID-19 continues to affect individuals disproportionately after infection with COVID-19. High education attainment was consistently associated with protective effects against post-COVID-19 condition in all our models as compared with low education attainment. This result is consistent with a large cross-sectional study in the USA with more than 16 000 respondents which reported less likelihood of persistent symptoms among individuals with high education.^24^ Education attained at an earlier age affects one’s health outcomes over the life-course. Level of education attainment attributes to working conditions, income and health, and therefore, is considered as the primary determinant of health disparities.^25^ In a large multinational population-based study of the effects of education levels and wealth on healthy ageing, in which the SHARE data were also used, Wu *et al* 26 found that the healthy ageing score declined with lower education levels and wealth.

Higher prevalence of obesity is associated with lower educational attainment.^27 28^ Our finding that BMI of individuals with high education attainment was significantly lower than that of individuals with lower education attainment suggests social inequality in health among older adults. Our analysis of comorbidity in relation to BMI showed a significant difference in BMI of people without any underlying health condition (26.5 kg/m^2^) as compared with one or more comorbidities (≥27.6 kg/m^2^). BMI increased with the number of comorbidities. The fact that high educational attainment and healthy weight were associated with lower likelihood of experiencing post-COVID-19 conditions, and that BMI was associated with educational attainment and the number of comorbidities in our study population may suggest that education attainment at earlier life affects one’s health through the life-course. COVID-19 exacerbated existing food insecurity.^29^ Poor diet quality can lead to obesity,^30^ and individuals with low diet quality living with high socioeconomic deprivation had the greatest risk of severe COVID-19 outcomes.^31^ The complex interactions between obesity, comorbidities, social determinants of health and post-COVID-19 condition observed in our study can be understood in the context of a new form of global syndemic of obesity; the COVID-19 syndemic of obesity, in which health disparities are exacerbated by synergetic interactions between poor health outcomes among people with obesity due to COVID-19, food insecurity caused by the pandemic, malnutrition and pre-existing social inequalities.^32^

In our study, non-V4 countries had protective effects for post-COVID-19 condition more than twice higher than V4 which is part of the Central and Eastern European Countries (CEEC). Earlier studies showed that postcommunist transition in 1990s adversely impacted income as well as education inequalities in CEEC, resulting in the rise in mortality rates among individuals with low education.^23 33^ In a study of 22 European countries, inequality in mortality associated with low education attainment was much higher in CEEC, and high education attainment was associated with lower mortality rates between 1990s and early 2000s.^23^ In a more recent study that examined the trend in mortality rates associated with education attainment between 1980 and 2014 in 27 European countries, inequality in mortality rates associated with low education and high education attainment remained higher in CEEC as compared with western Europe.^34^ This may suggest that greater health inequality derived from education attainment continues to affect health of individuals in Eastern European regions even after the postcommunist transition.^35^ WHO European Regional Obesity Report 2022 reports that the Eastern European regions have the highest overweight and obesity prevalence.^35^ Our analysis showed an association between regions and education attainment; the ratio of individuals with high education attainment was higher in non-V4 (23%) than V4 (10%), and furthermore, prevalence of obesity was higher in V4.

The present study indicated protective effects of COVID-19 vaccination against post-COVID-19 condition. The SHARE survey did not ask how many COVID-19 vaccines were administered and whether people were vaccinated before infection. We can assume, based on the European Union (EU) vaccine deployment plan in 2020,^36^ that only the first dose of vaccine was available before and during the survey period. Studies on effectiveness of vaccination(s) against post-COVID-19 condition showed varied rates of development of post-COVID-19 condition depending on the number of vaccines administered and when the vaccine was administered, that is, before or after infection. While we cannot draw a conclusion from the survey whether vaccination against post-COVID-19 condition is effective either before or after infection, at least one COVID-19 vaccination administered either before or after infection has been shown to reduce a likelihood of having post-COVID-19 condition or reduce the number of post-COVID-19 symptoms.^37–39^

### Strength and limitations

The strength of our study is that the SHARE survey is the largest pan-European longitudinal study of people aged 50 and above, and collected in a harmonised way across 28 European countries and Israel. However, our study has some limitations. SHARE is a self-reported survey, and information on COVID-19 test results, vaccination status and comorbidity was not confirmed by official health authorities. There was no question related to the number of vaccinations administered prior to the survey. As described earlier, we assumed that the initial dose of vaccination had been administered. The definition and symptom classifications of post-COVID-19 condition were announced by WHO after the survey period ended in August 2021. According to the current definition by WHO, post-COVID-19 condition is either lingering, or new symptoms that develop approximately 3 months after the infection and last for at least 2 months.^40^ There was no question in the survey about the duration of lingering conditions respondents experienced after the acute phase of COVID-19 infection. Post-COVID-19 condition addressed in the survey included eight symptoms, yet an increasing number of symptoms have been recognised as post-COVID-19 symptoms since the survey. There may be under-reported cases in which respondents did not consider that his or her condition was due to COVID-19 while the condition could have stemmed from other health issues. The present study did not explore race (ethnicity) or how racial bias may have affected post-COVID-19 condition, particularly how the severity of disease could have been detected early as possible. The accuracy of pulse oximeters provides such an example of racial bias in which skin colour was found to overestimate oxygen saturation of individuals with darker skin among black and Hispanic population in the USA, resulting in delayed treatment and increased risks of hypoxia.^41 42^

## Conclusions

Our findings highlight possible links to post-COVID-19 condition and health inequality associated with obesity and education attainment in older adults. Living outside V4 was a protective factor for post-COVID-19 condition. Health inequality was particularly evident in individuals in V4 with higher obesity prevalence and lower high education attainment than those living in non-V4 in the study. Our results suggest that education attainment earlier in life could affect one’s health in later years. We cannot draw causal inferences from the survey data; however, our findings suggest the need for obesity prevention, and further research is needed to understand persistent disparities in health inequality in European regions.

## Data Availability

Data are available in a public, open access repository.
